# Impact of extra-articular pathologies on groin pain: An arthroscopic evaluation

**DOI:** 10.1371/journal.pone.0191091

**Published:** 2018-01-11

**Authors:** Mitsunori Kaya

**Affiliations:** Hitsujigaoka Hospital, Sapporo, Hokkaido, Japan; Charles P. Darby Children’s Research Institute, 173 Ashley Avenue, Charleston, SC 29425, UNITED STATES

## Abstract

**Purpose:**

For patients who have anterior hip pain evaluated by Patrick’s test and tenderness at Scarpa’s triangle, we perform periarticular debridement based on the hypothesis that extra-articular pathologies are responsible for the hip pain. The purpose of this study was to categorize the endoscopic extra-articular findings and to evaluate the clinical significance of periarticular pathologies in anterior hip pain.

**Methods:**

Arthroscopic findings of 77 patients who underwent periarthritic debridement were evaluated. As extra-articular pathologies, injuries of the direct head and reflective head of the rectus femoris muscle were evaluated. A thin layer of fat tissue normally exists on the anterior inferior iliac spine (AIIS), the attachment site of the direct head of the rectus femoris muscle. The macroscopic appearance of the fat pad on the AIIS was categorized as normal, blood vessel-rich adipose tissue or adipose tissue with fibrosis or scar formation and histologically confirmed. Adhesion of gluteal muscles to the joint capsule was also evaluated.

**Results:**

Of the 77 patients, 75 had rupture of the direct head of the rectus femoris. In contrast, rupture of the reflective head was extremely rare. Seven patients had a normal fat pad on the AIIS, 11 had blood vessel-rich adipose tissue and 55 had adipose tissue with fibrosis. Fat tissue was completely replaced by fibrous scar tissue in another 4 patients. In 64 patients, adhesion between the anterior joint capsule and gluteus muscles was marked. Groin pain disappeared soon after the operation even when labral tears were not repaired and all patients returned to daily life and sports activities within 2 weeks after operation.

**Conclusion:**

Rectus femoris tendinosis, fibrosis of the AIIS fat pad, and adhesion of gluteal and rectus femoris muscles are common extra-articular pathologies in patients with anterior hip pain. Management of only these lesions induces rapid relief of anterior hip pain even in the absence of labral tear repair. My observations suggest that it is desirable to be aware of the presence of periarticular pathologies as a cause of groin pain.

## Introduction

Groin pain is a common problem and is known to be a complex issue [1]. The wide variety of possible pathologies in numerous anatomical structures contributes to this complexity [2]. Intra-articular pathologies like acetabular labrum tears, cartilage damage and ligamentum teres tears are believed to be major problems causing hip pain. Currently, FAI correction and labral repair are common therapeutic modalities for the management of groin pain [3,4]. In contrast, extra-articular pathologies like tendinosis of the direct head of the rectus femoris and bursitis around the hip joint receive relatively little attention as targets of surgical intervention.

Patrick’s test is a manual examination to evaluate the pathology of the hip joint [5]. This test is performed by flexing the tested leg and abducting and externally rotating the thigh. If pain is elicited in the hip joint, it is suggestive of a hip joint disorder. It is well known that Patrick’s test involves several pain sites. The variety of pain sites depends on the structures damaged and understanding this is necessary for correct diagnosis and treatment planning. For patients who have anterior hip pain in Patrick’s test and tenderness at Scarpa’s triangle, we perform periarticular debridement based on the hypothesis that periarticular pathologies are responsible for the hip pain. In the current manuscript, I demonstrate endoscopic extra-articular findings to evaluate the clinical significance of periarticular pathologies. The hypothesis is that periarticular pathologies are common in patients with groin pain and more severe than we normally think.

## Materials and methods

Permission to conduct research involving human participants was obtained through the appropriate institutional review board. All participants were informed of the purpose of the study, and they provided written informed consent before participating. It is well known that Patrick’s test can reveal several pain sites: anterior, lateral and posterior. The pain site is defined based on the structures damaged. I have developed a hypothetical diagnostic algorithm for hip pain and decide the treatment option according to this algorithm. If patients feel hip pain anteriorly in Patrick’s test and have tenderness at Scarpa’s triangle, tendinosis of the direct head of the rectus femoris muscle is suspected. Ultrasound-assisted lidocaine injection into the region of the anterior inferior iliac spine (AIIS) is helpful to make a diagnosis of anterior periarthritis. If patients feel surface lateral hip pain in Patrick’s test and have tenderness over the greater trochanter, greater trochanter bursitis is suspected. Pain relief is confirmed by lidocaine injection into the greater trochanter bursa. If patients feel deep lateral discomfort, we suspect a labral tear and confirm it by lidocaine injection into the hip joint.

**Fig 1:**
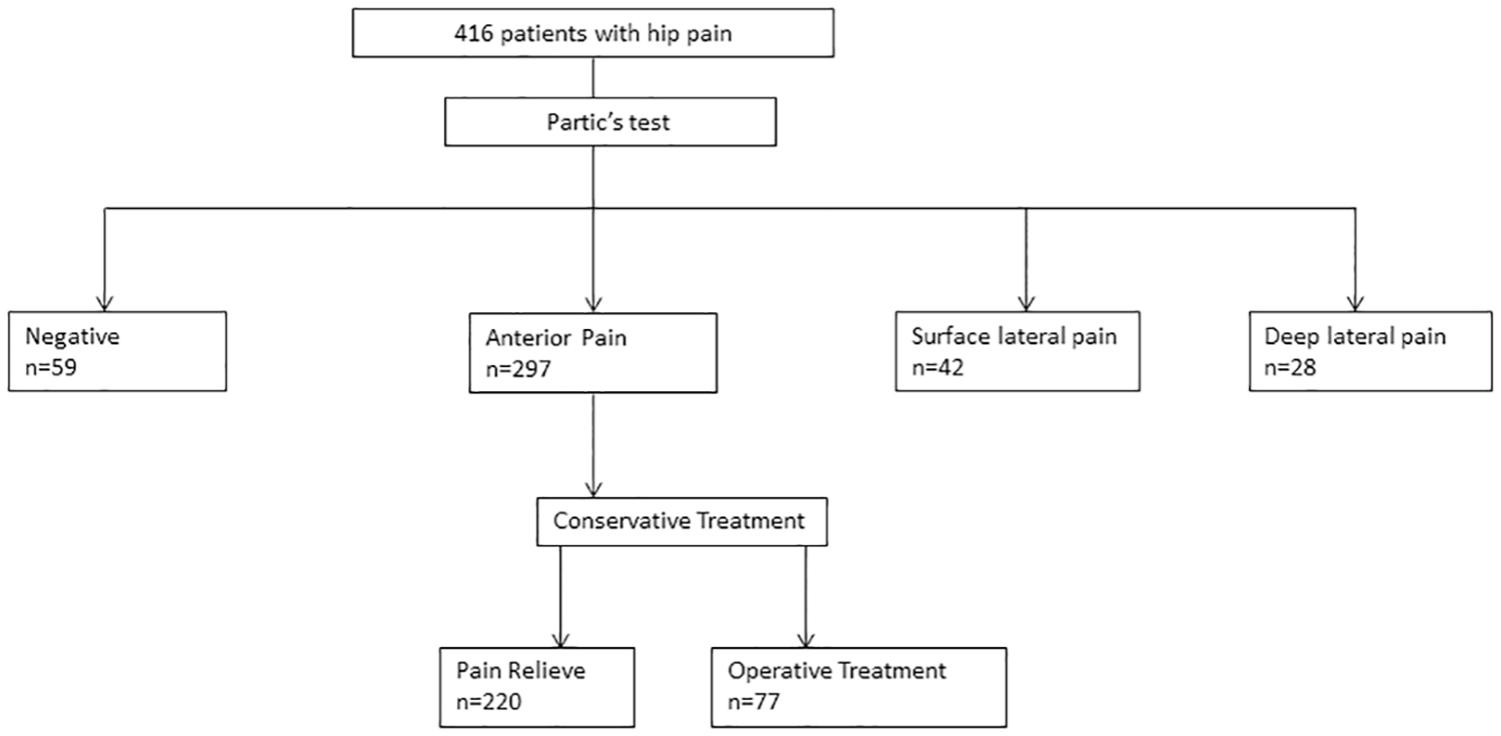
Flowchart of diagnosis based on the pain site of Patric test. Diagnoses were determined according to the location of the pain in Patric test.

Since April 2016, 416 patients with hip pain have visited to our clinic. Of these, 297 had anterior hip pain in Patrick’s test and tenderness at Scarpa’s triangle. The diagnosis of anterior periarthritis of the hip joint was confirmed by ultrasound guided lidocaine injection into the region of the AIIS. After the diagnosis of anterior periarthritis, all patients underwent physical therapy for at least 6 weeks and symptoms were relieved in 220 patients (74.0%). Surgical treatment was carried out for the remaining 77 patients who were resistant to the conservative therapy and they were included to this study (Fig 1).

**Fig 2:**
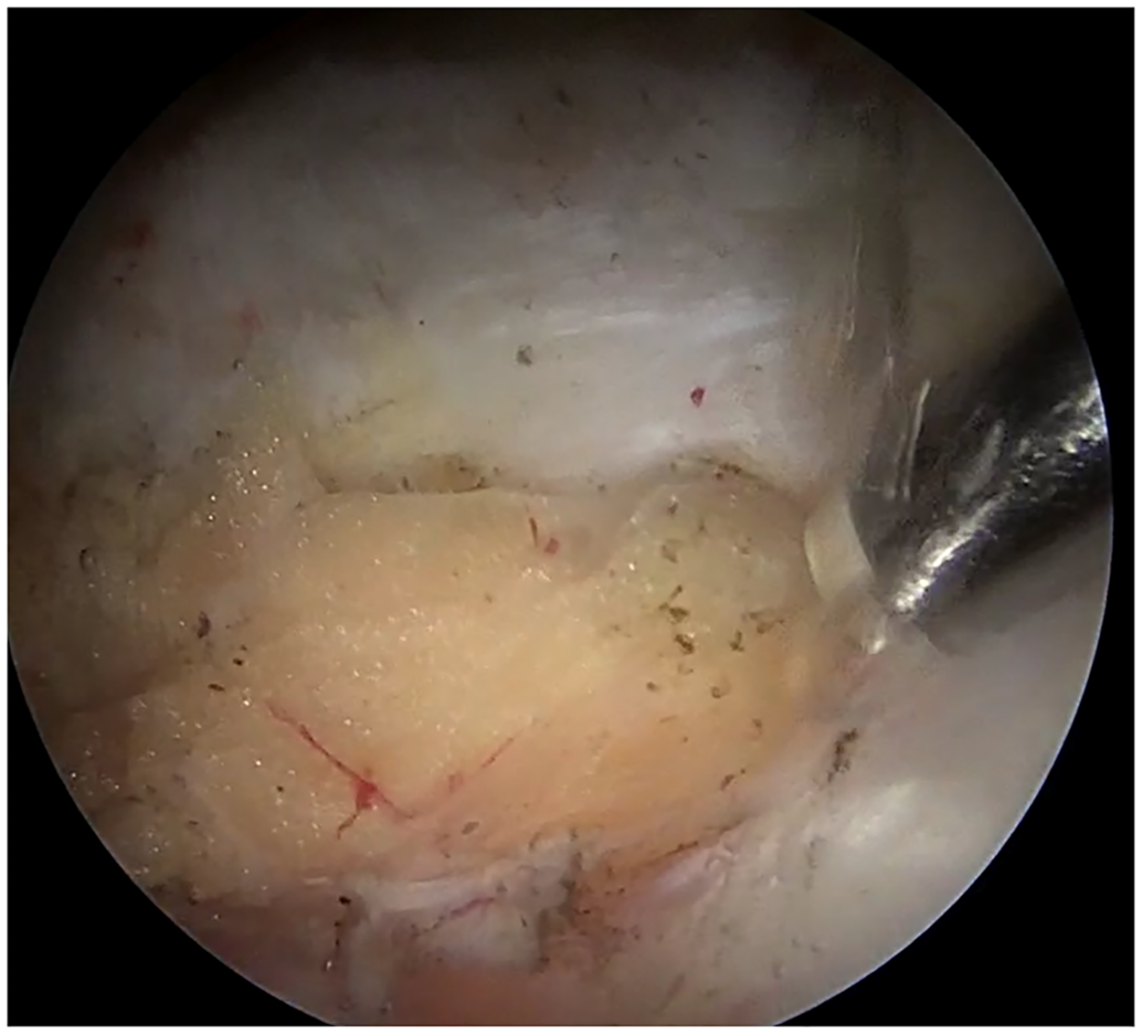
Screenshot from the video of a normal fat pad on AIIS in the right hip viewing from anterolateral portal. Thin layer of fat tissue normally exists on the AIIS and anterior joint capsule.

**Fig 3:**
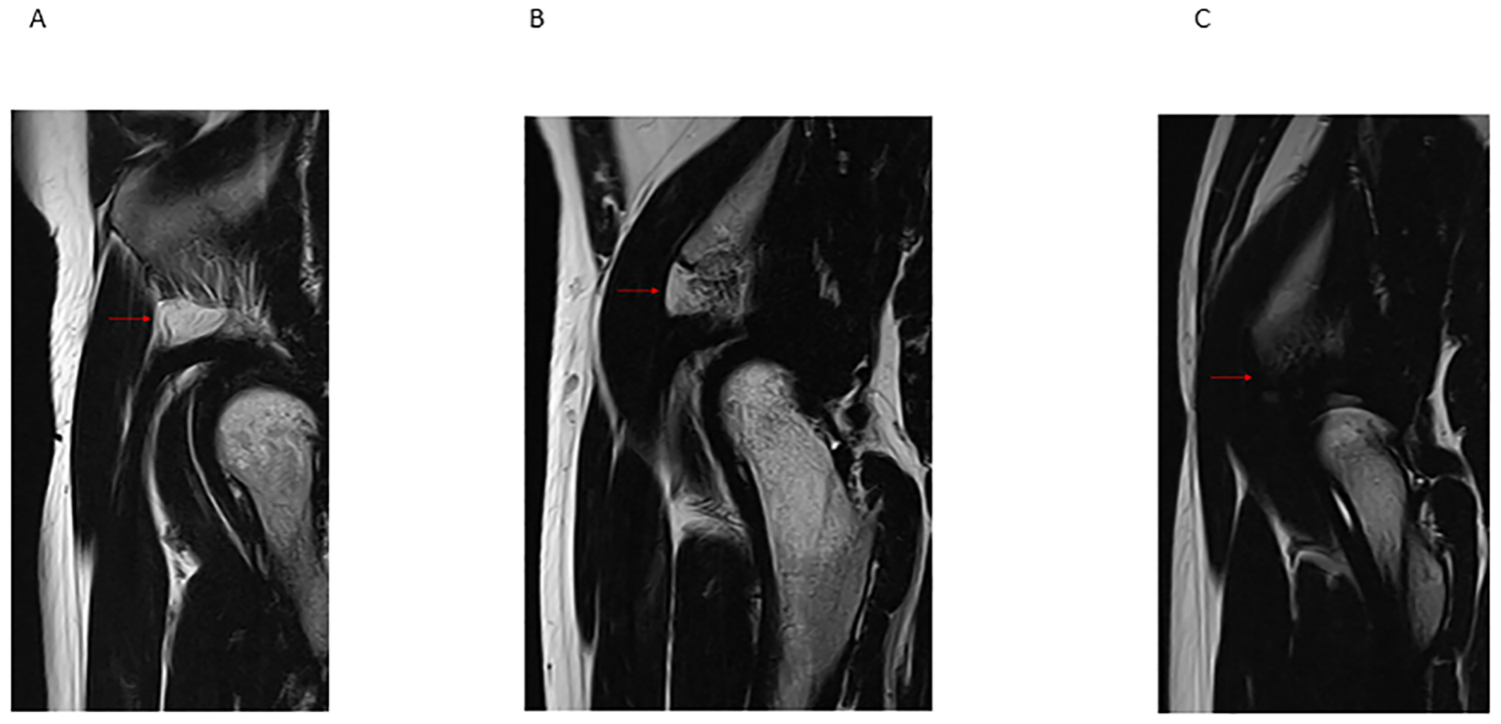
T2-weighted sagittal image of hip joint. Normal fat pad (A, red arrow), fat pad with fibrosis (B, red arrow), fibrous scar formation (C, red arrow).

Those with radiographic parameters consistent with FAI (crossover sign, pistol grip deformity, and an alpha angle greater than 55°) were diagnosed as having FAI [6,7]. The patients were diagnosed with acetabular dysplasia if the lateral center-edge angle of Wiberg (LCEA) was less than 25° [8] and with borderline dysplasia if the LCEA was between 20° and 25° [9]. A thin layer of fat tissue normally exists on the AIIS and anterior joint capsule (Fig 2 & S1 Movie). On sagittal MR imaging, the status of this thin fat layer was evaluated. On T1- and T2-weighted MRI, the appearance the AIIS fat pat is similar to the surrounding subcutaneous fat (Fig 3A). The presence of additional lower signal foci represents septal fibrosis (Fig 3B). Increased T1- or T2-hypointense signals within the fat pad can indicate massive fibrosis (Fig 3C). The presence of increased signal foci of the tendon seen on coronal MRI indicates rupture of the direct head of the rectus femoris muscle at the insertion of the AIIS.

All the surgical procedures were performed with the patient in the supine position using a traction table. Routine diagnostic arthroscopy was performed to assess the ligamentum teres, cartilage surfaces, and labrum. As I hypothesized that intra-articular pathologies were not responsible for the hip pain in this cohort, we never repaired the injured labrum. We next assessed the extra-articular lesions, direct head and reflective head of the rectus femoris muscle, the thin fat pat on the AIIS and anterior joint capsule. Any torn fibers of the direct head of recuts femoris muscle were debrided with a shaver and electrocautery, leaving healthy intact fibers. The AIIS fat pad was also debrided with a shaver and electrocautery and bony protrusions of the AIIS were decorticated with a bone cutting bar. Formal physical therapy was begun on postoperative day 1 without any restriction of weight bearing or the range of motion. Patients were returned to the normal activities of daily life and sports activities 2 weeks after the operation.

**Fig 4:**
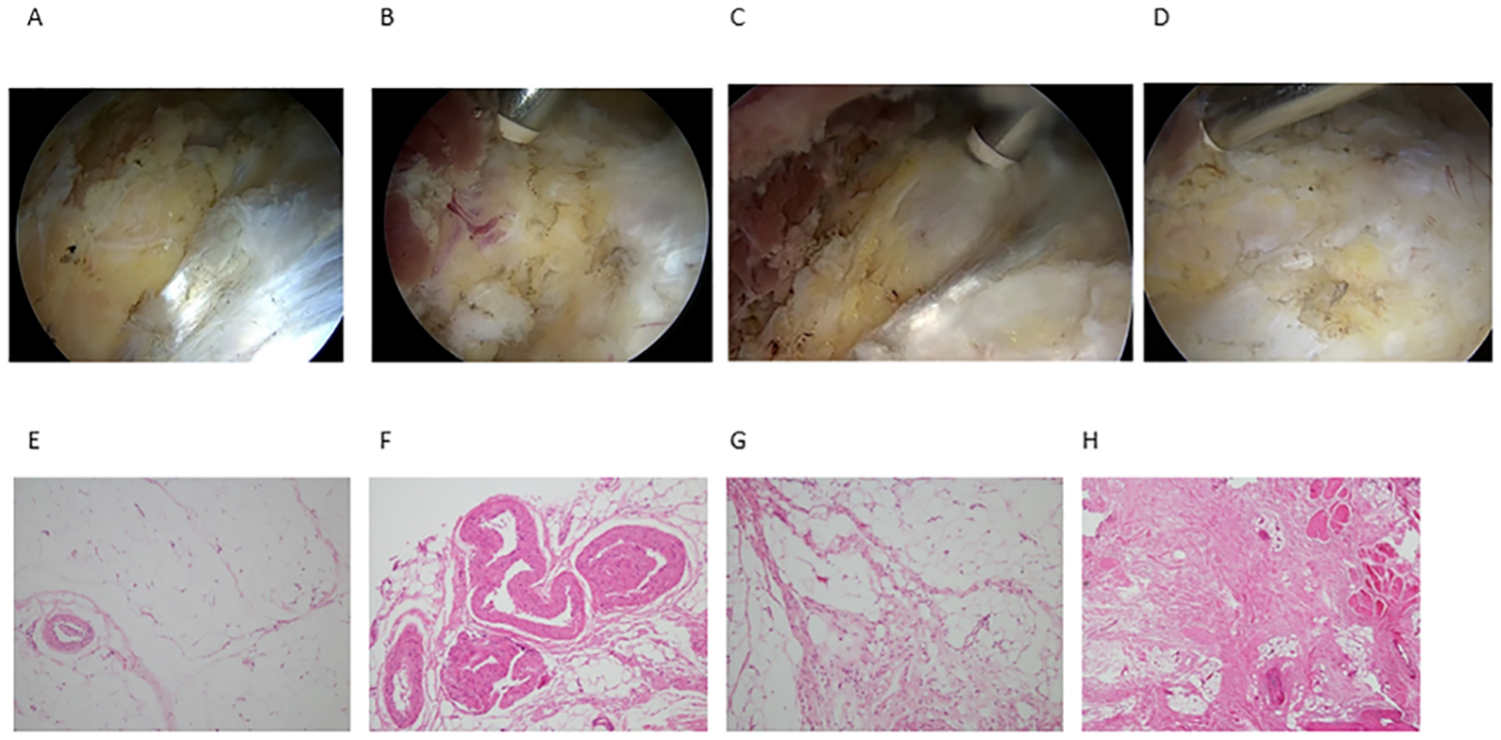
Screenshot from the video and histology of AIIS fat pad. Normal fat fad (A, E), blood vessel-rich adipose tissue (B, F), fat pad with septal fibrosis(C, G), fat pad replaced by fibrous scar (D, H).

Ligamentum teres tears were classified according to the system of Gray and Villar [10]. The patterns of labral tears were divided into main groups analogous to the classification system described by Lage [11]. Partial or complete injury of the direct head or reflective head of the rectus femoris muscle was arthroscopically evaluated. The macroscopic appearances of the fat pad on the AIIS and anterior joint capsule were categorized as follows: normal fat tissue, blood vessel rich-adipose tissue, and adipose tissue with fibrosis and scar formation (Fig 4 & S2 Movie). The fat pad on the AIIS was resected, and fixed in 10% formalin for 24 hours and then embedded in paraffin. The embedded samples were cut into 5 μm slices, and placed on saline-coated glass slides, with standard H & E protocols used for sample staining. Histological evaluation was performed by a pathologist.

Outcomes were evaluated for all hips using the modified Harris Hip Score (mHHS) [12] and the visual analogue scale (VAS) (a scale of 0 to 10 with 0 = no pain, and 10 = very bad pain).

Statistical analyses were performed using IMB SPSS Statistics 22.0 (IBM SPSS Inc. USA). The mean and standard deviation were used for descriptive statistics with 95% confidence intervals (CI). Pre- and postoperative scores were compared using the paired *t*-test.

## Results

**Fig 5:**
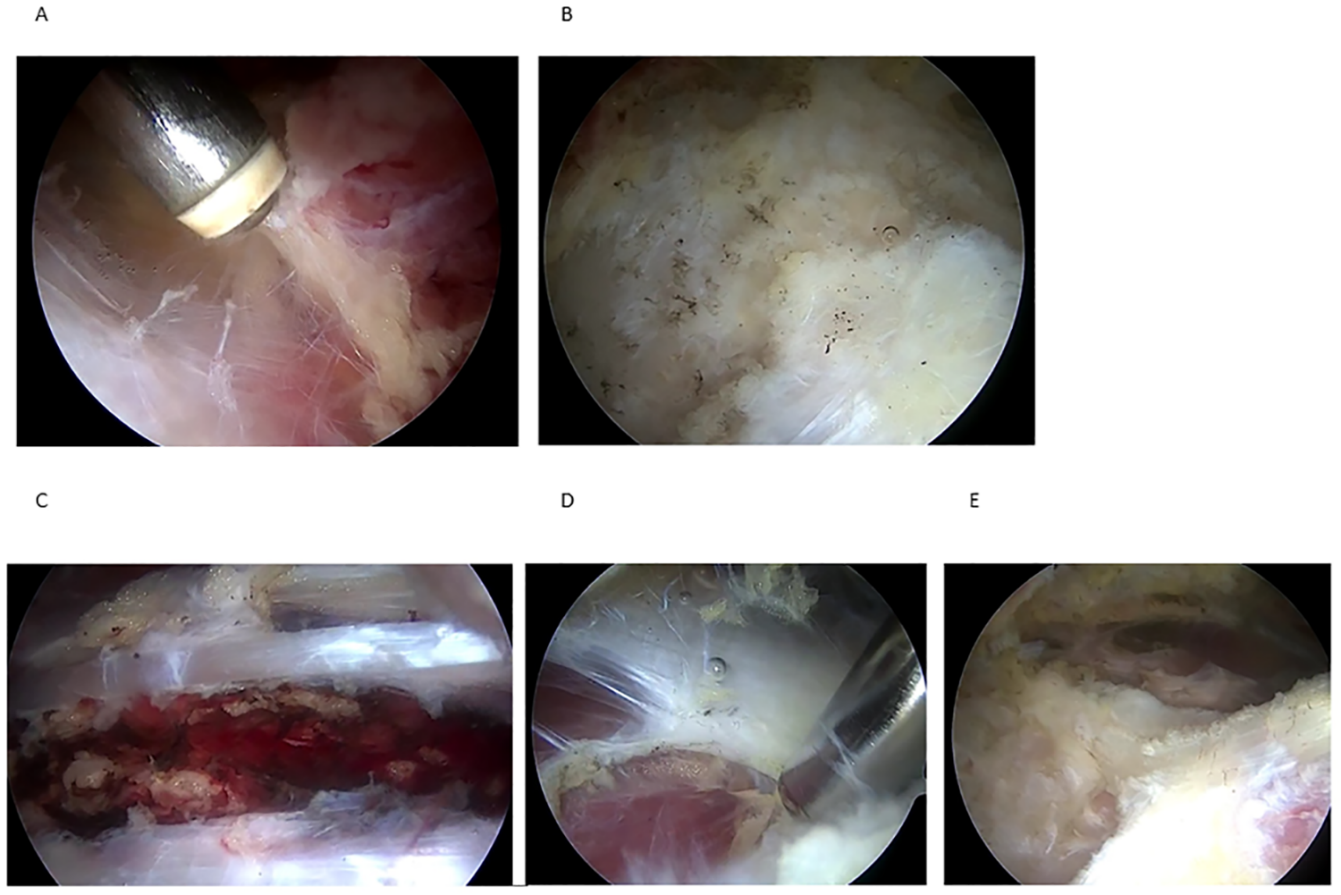
Screenshot from the video of periarticular area in the right hip viewing from anterolateral portal. Adhesion between anterior joint capsule and gluteus muscles(A), Rupture of direct head of rectus femoris muscle at the insertion to AIIS(B), Neovascularized mature fat tissue between rectus femoris muscle and iliocapsularis muscle(C), Iliopectoralis bursitis (D), periarthritic view after the completion of debridement (E).

The patient population consisted of 27 male and 50 female patients with a mean age of 38.1±19.7 years (range, 13–78 years, 95%CI 33.4 to 42.9). Their demographic data are shown in Table 1. The radiological diagnoses were femoroacetabular impingement (FAI) in 28 (cam, 2; pincer, 23; mixed, 3), acetabular dysplasia in 8, borderline dysplasia in 17, osteoarthritis in 4, and normal in 20. Of the 77 patients, 72 (93.5%) had positive anterior impingement signs. A thin layer of fat tissue normally exists on the AIIS. The status of this fat pad was evaluated by sagittal MR imaging. Nine patients had the normal fat pad signals, 64 had a normal high signal fat pad with additional lower signal foci suggesting septal fibrosis, 4 had increased T1- and T2-hypointense signals within the fat pad, indicating massive fibrosis and scar formation, and 68 had increased signal foci of the tendon at the insertion of AIIS, indicating tendinosis of the direct head of the rectus femoris muscle (partial in 53, and complete in 15). Regarding the intra-articular pathologies, 67 patients had acetabular labral tears, 14 had delamination, 53 had rupture of the labro-chondral junction and 52 had ligamentum teres tears (complete tears in 20 and partial ruptures in 32) (Table 2). Details of the periarticular pathologies are shown in Fig 5, Table 2 and S3 Movie. There were 75 patients with tendinosis of the direct head of the rectus femoris muscle (partial injury in 56 and complete rupture in 19). In contrast, rupture of the reflective head was extremely rare. Only 7 patients had partial ruptures of the reflective head. Another 7 had a normal fat pad on the AIIS (Fig 4A & 4E). There were 11 patients with blood vessel-rich adipose tissue. The gross findings were yellow, vascular-rich mature adipose tissue (Fig 4B). Histologically, mid-sized to large vessels were observed within the adipose tissue (Fig 4F). There were 55 patients who had adipose tissue with fibrosis. Macroscopically, the fat pad was changed to whitish fibrous fat tissue (Fig 4C). Histologically, adipose tissues with septal fibrosis and mild infiltration of inflammatory cells into the septa were observed (Fig 4G). Some adipocytes had myxoid degeneration suggestive of disruption of the microcirculation. Fat tissue was completely replaced by fibrous scar tissue in another 4 patients (Fig 4D). Microscopically, most of the normal fat tissue was replaced by fibrous scar tissue (Fig 4H). In some cases, inflammatory reactions were marked in the fat pad between the rectus femoris muscle and iliocapsularis muscles. There were 40 patients who had hemorrhage and 12 had neovascularization in intermuscular fat pads. In 64 patients, adhesion between the anterior joint capsule and gluteus muscles was obvious.

**Table 1:** Patient’s demographics.

Gender	Male	27
	Female	50
Ave. age	38.1±19.7(13–78 years, 95%CI 33.4 to 42.9)	
Radiological diagnoses	Normal	20
	FAI	28
	acetabular dysplasia	8
	borderline dysplasia	17
	osteoarthritis	4

**Table 2:** Intra- and extra-articular pathologies.

Intra-articular pathologies		
Labral tear	Intact	10
	Delamination	14
	labro-chondral junction injury	53
Ligamentum teres	Intact	25
	partial tear	32
	complete tear	20
Extra-articular pathologies		
Direct head of rectus femoris m.	intact	2
	partial injury	56
	complete rupture	19
Reflective head of rectus femoris m.	intact	70
	partial injury	7
Fat pad on AIIS	normal	7
	blood vessel-rich adipose tissue	11
	adipose tissue with fibrosis.	55
	Fibrous scar	4
Fat pad between rectus femoris and iliocapsularis	Intact neovascularization hemorrhage	25 40 12
Adhesion between anterior joint capsule and gluteus muscles	No Yes	13 64

Based on the hypothesis that not intra-articular pathologies but rather periarticular lesions around the AIIS are responsible for the groin pain in patients who have anterior hip pain in Patrick’s test, only periarticular debridement was performed. We never repaired the labrum. Neither cam decompression for FAI nor acetabuloplasty for acetabular dysplasia was performed. The procedure included arthroscopic debridement of the torn direct head of the rectus femoris and fibrotic fat pad using a shaver or electrothermal probe. Adhesions of muscles to the joint capsule were also released. Cauterization of any visible vascularization in the diseased tissue completed the procedure. Groin pain disappeared soon after the operation even when the torn labrum was not repaired. All patients returned to their daily life and sports activities within 2 weeks after operation. They were all followed-up with a mean follow-up period of 8.3 months (range, 16 to 7 months). There were significant improvements in the postoperative mHHS and VAS (Table 3).

**Table 3:** Pre- and post-operative clinical score.

Outcome Measure	Pre-OP	Post-OP	*P* value	*95%CI*
mHHS	68.7±10.7	97.3±2.8	< .001	-31.1 to -26.1
VAS	5.4±1.1	1.5±3.0	< .001	3.1 to 4.6

## Discussion

I have presented the endoscopic findings in patients with anterior hip pain confirmed by Patrick’s test. In addition to intra-articular pathologies, all patients had periarticular pathologies. Tendinosis of the direct head of the rectus femoris muscle was common and fibrosis of the fat pads on the AIIS and anterior joint capsule was more severe than we originally thought.

MR images showed the fibrosis of the thin AIIS fat pad. I also confirmed this lesion histologically. However, the mechanism of the injury of the direct head of the rectus femoris muscle remains unclear. Based on the macroscopic and histologic findings, I speculate that this is not a simple trauma but a repetitive degenerative process. Repetitive eccentric and concentric stresses may cause microtears of the tendons and a reactive ischemic gelatinous response that spreads to the adjacent fat pad, resulting in inflammatory cell infiltration and myxomatous degeneration of adipocytes. The prolonged and persistent reaction would lead to the production of fibrous tissue and result in the formation of fibrous scar tissue and adhesion of the muscles. In our case series, the majority of the lesions were composed of fibrosis and scar tissue formation. I think that tendinosis of the rectus femoris tendon and chronic fibrous degeneration of adipose tissue are major pathologies of patients with recalcitrant anterior hip pain. As with tendinosis of the ECRB in tennis elbow, I assume the same pathology as AIISpinitis, even in the hip joint [13,14].

The technical and instrumental development of hip arthroscopic surgery enabled us to treat intra-articular pathologies. Most clinicians focus on the acetabular labrum as the target lesion of hip pain. Hip impingement surgery is now common and satisfactory mid-term clinical results have been reported by many institutions [15]. However, this may be a biased idea because it is very difficult to confirm the real effect of labral repair due to the lack of comparative studies with adequate controls. The simple question of what a positive anterior impingement test means in patients with tenderness at Scarpa’s triangle remains. Are acetabular labral tears responsible for the anterior hip pain in such patients? This was one of the triggers that led us to develop the current hypothesis [16]. Most of the patients in this cohort had positive anterior impingement signs and I confirmed that they had labral tears arthroscopically. In such a situation, most surgeons generally believe that labral tears are the cause of hip pain and labral repair is commonly performed for the management of this pain. However, I performed only periarticular debridement in accordance with our hypothesis. The hip pain was relived rapidly soon after the operation even without labral repair. I believe this finding strongly indicates that periarticular pathologies can also be major causes of groin pain.

This study has several limitations. First, it was a simple observational study with a small sample size. At present, normal anatomy of this periarticular area is not well understood. To confirm my hypothesis, a comparative study with asymptomatic patients is necessary. It is critical to make a preoperative diagnosis of the injury of the direct heads of rectus femoris muscle. However, MRI cannot detect it clearly. This is also a limitation of this manuscript. The establishment of the MRI imaging method to detect the tendinosis of the direct head of rectus femoris muscle is mandatory. Due to the very short follow-up periods, it is premature to decide the clinical effectiveness of periarticular debridement for the management of groin pain. Therefore, I have no intention to argue this point. Even if these are very early results, however, the fact that hip pain was relieved soon after the operation can provide strong evidence for the determination of extra-articular pathologies being responsible for groin pain. I think this is a strong point of this manuscript.

## Conclusions

Rectus femoris tendinosis, subacute /chronic fibrosis of the fat pad and muscle adhesion are common extra-articular pathologies in patients with anterior hip pain. In addition, management of these lesions results in rapid relief of the hip pain. Current observations suggest that it is desirable to be aware of the presence of periarticular pathologies as a cause of groin pain.

## Supporting information

S1 Movie Arthroscopic video showing a normal fat pad on AIIS in the right hip viewing from anterolateral portal. Thin layer of fat tissue was normally exists on the AIIS and anterior joint capsule.
(MP4)

S2 Movie Arthroscopic video showing an AIIS fat pad pathogies. Normal fat fad), blood vessel-rich adipose tissue, fat pad with septal fibrosis, fat pad replaced by fibrous scar were shown.
(MP4)

S3 Movie Arthroscopic video showing a periarticular pathologies in the right hip viewing from anterolateral portal. Adhesion between anterior joint capsule and gluteus muscles, rupture of direct head of rectus femoris muscle at the insertion to AIIS, neovascularized mature fat tissue between rectus femoris muscle and iliocapsularis muscle and Iliopectoralis bursitis were shown.
(MP4)
